# Age-related decline in bone mineral transport and bone matrix proteins in osteoblasts from stromal stem cells

**DOI:** 10.1152/ajpcell.00227.2023

**Published:** 2023-07-31

**Authors:** Irina L. Tourkova, Quitterie C. Larrouture, Kelechi M. Onwuka, Silvia Liu, Jianhua Luo, Paul H. Schlesinger, Harry C. Blair

**Affiliations:** ^1^Research Service, VA Medical Center, Pittsburgh, Pennsylvania, United States; ^2^Department of Pathology, University of Pittsburgh, Pittsburgh, Pennsylvania, United States; ^3^Department of Cell Biology & Physiology, Washington University in St. Louis, St. Louis, Missouri, United States

**Keywords:** aging, bone mineralization, osteoblast, osteoporosis, stromal stem cells

## Abstract

We studied osteoblast bone mineral transport and matrix proteins as a function of age. In isolated bone marrow cells from long bones of young (3 or 4 mo) and old (18 or 19 mo) mice, age correlated with reduced mRNA of mineral transport proteins: alkaline phosphatase (ALP), ankylosis (ANK), the Cl^−^/H^+^ exchanger ClC3, and matrix proteins collagen 1 (Col1) and osteocalcin (BGLAP). Some proteins, including the neutral phosphate transporter2 (NPT2), were not reduced. These are predominately osteoblast proteins, but in mixed cell populations. Remarkably, in osteoblasts differentiated from preparations of stromal stem cells (SSCs) made from bone marrow cells in young and old mice, differentiated in vitro on perforated polyethylene terephthalate membranes, mRNA confirmed decreased expression with age for most transport-related and bone matrix proteins. Additional mRNAs in osteoblasts in vitro included ecto-nucleotide pyrophosphatase/phosphodiesterase 1 (ENPP1), unchanged, and ENPP2, reduced with age. Decrease with age in ALP activity and protein by Western blot was also significant. Transport protein findings correlated with micro-computed tomography of lumbar vertebra, showing that trabecular bone of old mice is osteopenic relative to young mice, consistent with other studies. Pathway analysis of osteoblasts differentiated in vitro showed that cells from old animals had reduced Erk1/2 phosphorylation and decreased suppressor of mothers against decapentaplegic 2 (Smad2) mRNA, consistent with TGFβ pathway, and reduced β-catenin mRNA, consistent with WNT pathway regulation. Our results show that decline in bone density with age reflects selective changes, resulting effectively in a phenotype modification. Reduction of matrix and mineral transport protein expression with age is regulated by multiple signaling pathways.

**NEW & NOTEWORTHY** This work for the first time showed that specific enzymes in bone mineral transport, and matrix synthesis proteins, in the epithelial-like bone-forming cell layer are downregulated with aging. Results were compared using cells extracted from long bones of young and old mice, or in essentially uniform osteoblasts differentiated from stromal stem cells in vitro. The age effect showed memory in the stromal stem cells, a remarkable finding.

## Graphical abstract

**Figure:**
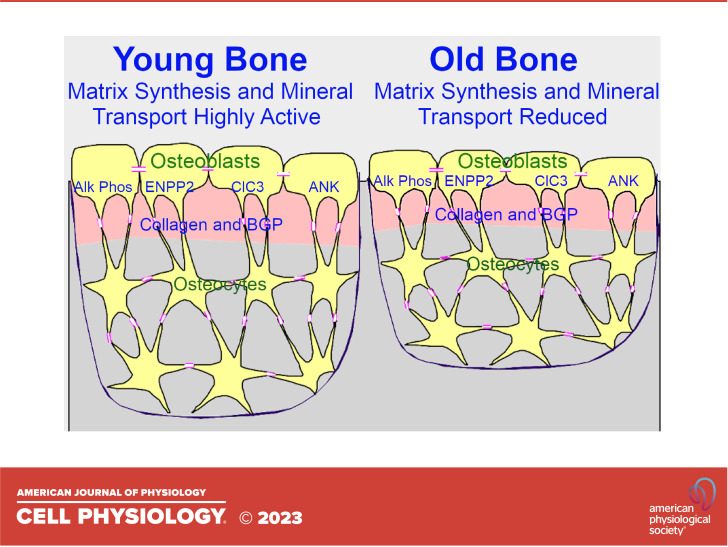

## INTRODUCTION

Age-related bone loss and increased risk for bone fractures in the elderly population are established (1–3). Peak bone mass in humans is at 25–30 yr, or 4–6 mo in mice; osteopenia is observed in people over 50 yr, or ∼18 mo in mice. At older ages, the amount of bone produced by osteoblasts is less than osteoclast bone resorption. We investigate the uncoupling of formation from resorption in older animals.

Multiple studies describe age-related changes in bone mechanical function and bone matrix composition (2), but underlying transport mechanisms essential for production of mineralized bone are largely not studied as a function of age. Our purpose is to study the expression of enzymes that mediate mineral accumulation and to correlate this with changes in bone matrix formation. We studied this using cells flushed from bone marrow of young and old mice, and from isolated stromal stem cells (SSCs; also called skeletal stem cells), which were used to prepare osteoblasts differentiated in vitro, which, remarkably, captured the effects of age on mineralization and of changes in regulatory proteins for differentiation.

Specifically, our previous work, and that of others, shows that bone components are synthesized by transport across an epithelial-like layer of osteoblasts connected by tight and gap junctions (4–7).

Important related findings include that living bone does not allow passive transport of calcium-binding fluorescent indicators across its surface, whereas dead bone accumulates calcium-binding fluorescent indicators passively (8). The epithelial-like surface is reproduced in vitro as a cell layer of osteoblasts with epithelial resistance using stromal stem cells on perforated polyethylene terephthalate (PET) membranes (9). We added this source of young and old osteoblasts to direct analysis of marrow cells to assure results without any interference from marrow cells or osteoclasts.

In bone synthesis, osteoblasts produce and secrete the major bone structural proteins, collagen 1 and osteocalcin (BGLAP). The extracellular collagen trimers and osteocalcin occur in layers at alternating right angles, forming extracellular matrix onto which mineral is added (7). This dense collagen matrix is mineralized as the osteoblasts provide calcium and phosphate and remove protons (5–7). Bone represents the large majority of the Ca^2+^, HPO_4_, and base-equivalents in the vertebrate body (7). This is important because bone mineral requires deposition of massive amounts of calcium, phosphate, and base equivalents. This is often not appreciated, but it is essential to bone mineralization (7):

(*1*) $$\text{6 HP}{{\text{O}}_{4}}^{2-}+\text{ 2 }{\text{H}}_{2}\text{O }+\text{ 1}0\text{ C}{\text{a}}^{2+}↔\text{ C}{\text{a}}_{10}{\left(\text{P}{\text{O}}_{4}\right)}_{6}{\left(\text{OH}\right)}_{2}+\text{ 8 }{\text{H}}^{+}.$$

Expression of mineral transport-related proteins with age is our subject. This was studied in cells from long bones of young and old mice, and in stromal stem cells from young and old mice differentiated in vitro to osteoblasts as epithelial-like surfaces on polyethylene terephthalate membranes (9).

The most abundant enzyme for mineral production has long been known to be the liver-kidney-bone alkaline phosphatase (ALP) (10). It catalyzes the phosphate ester hydrolysis reactions and the phosphate is incorporated into bone as summarized in *Eq. 1*. Its functions include pyrophosphate degradation (11) to the point that, in hypophosphatasia, very low ALP increases pyrophosphate, inhibiting mineralization (12). But, under normal circumstances, ALP is an abundant cell membrane-bound enzyme directly producing phosphate incorporated into bone, definitively demonstrated using osteoblasts from ALP knockout animals in vitro (13). That said, ALP has multiple functions and is expressed in many cells (14); our interest is solely in the cell membrane-bound form of ALP in osteoblasts. In addition to ALP, other enzymes that regulate phosphate production in osteoblasts were studied relative to age. These include ecto-nucleotide pyrophosphatase/phosphodiesterase (ENPP) family members (15), the ankylosis homology protein (ANKH), believed to regulate bone formation (16), and the type 2 sodium-phosphate cotransporter (NPT2), the major phosphate importer in osteoblasts (17). In vitro, NPT2 expression in osteoblasts increases with differentiation (17), but differences with osteoblast age were unknown. Other regulators of mineralization include, in vitro, Cl^−^/H^+^ antiporters ClC3 and ClC5 (5) and enzymes that transport acid equivalents out of osteoblasts. This role for ClC family genes in bone is unusual and controversial but is supported by our earlier in vitro work (5). In contrast, cytoplasmic pH regulation by Na^+^/H^+^ is demonstrated (6) with very active pH regulation by Na^+^/H^+^ exchangers NHE1 and NHE6 at the basolateral cell membrane.

To validate that the study reflects changes in bone mass with age similar to previous studies of aging and bone in mice, we determined the bone structure and matrix parameters in matched young and old mice by micro-computed tomography (microCT) and studied pathways regulating bone formation in young and old mice. These also show effects of age that are conserved in young and old SSCs.

## MATERIALS AND METHODS

### Animals

We used syngeneic C57BL/6J 3- to 4-mo-old mice from The Jackson Laboratory and C57BL/6J 18- to 19-mo-old mice, a kind gift from National Institutes of Health, National Institute on Aging, C57Bl/6J RRID: MGI:2159769. The same number of male and female mice was used in each age category; males and female animals in specific experiments are indicated in the figure legends. The animal protocol was approved by the University of Pittsburgh Institutional Animal Care and Use Committee.

### Media, Reagents, and Chemicals

Materials were from Thermo Fisher Scientific (Waltham, MA) or as stated. All media contained antibiotics and antimycotics.

### Isolation of Bone Cells

Cells from trabecular bone surfaces and bone marrow of mouse femur and tibia were flushed using 10-mL syringe with RPMI medium with 10% FBS. Erythrocytes were removed with cell lysis buffer. Half of stromal bone marrow cells were used directly for RNA isolation as in vivo analysis of mRNA expression; the other half were used for isolation of stromal stem cells. Additional preparations of bone marrow of mouse femur and tibia, from the same animal populations, were used for derivation of stromal stem cells only to provide the necessary number of preparations for data shown.

### Marrow-Derived SSCs

Cells are plated overnight to remove fibroblasts and then processed as described (18). In brief, nonadherent cells were replated in RPMI for 72 h, and medium was replaced with mesenchymal stromal cell culture expansion media (MesenCult, STEMCELL Technologies, Vancouver, BC, Canada). After SSCs grow well, MesenCult medium is changed to DMEM with 1.0 g/L glucose and 10% FBS (proliferation medium). For SSC differentiation into osteoblasts, cells at 90% confluence were placed in osteogenic differentiation medium (proliferation medium with 30 μg/mL l-ascorbic acid, 10 mM 2-phosphoglycerol, and total calcium concentration 2 mM). SSCs were differentiated for 2 wk. Medium is refreshed twice a week. The properties of cells were confirmed by flow cytometry for stem cell and control antigens as described (9). Those repeating this work are advised that, in our experience, the MesenCult medium (STEMCELL Technologies, Vancouver) is essential for achieving a high success rate of these preparations.

### Differentiation of Osteoblasts on Permeable Membranes

We produced osteoblasts in epithelium-like form and verified by electrical resistance (9). These were used for the real-time PCR and other analyses as described. In brief, SSCs were differentiated in osteogenic differentiation medium on polyethylene terephthalate (PET) membrane inserts with 0.4-μm perforations that optimizes mineral transport into the matrix and acid transport (9). Expression of osteoblast markers including alkaline phosphatase, collagen, and mineral in these preparations is documented (9). Differentiation was confirmed in each preparation by alkaline phosphatase and von Kossa mineral staining. Time for SSC isolation and differentiation was 2 mo.

### RNA Isolation and Real-Time PCR

Total RNA, isolated by oligo dT affinity (RNeasy, Qiagen, Hilden Germany), was reverse transcribed using random hexamers and reverse transcriptase (SuperScript III, Invitrogen, Thermo Fisher). Real-time PCR used an MX3000P instrument (Stratagene, San Diego, CA) with SYBR Green (Thermo Fisher) to monitor DNA synthesis. Reactions were run in duplicate, in 25 µL reaction volume with Brilliant III Ultra-Fast SYBR Green QPCT Master Mix (Thermo Fisher), 250 nM primers, and cDNA. After 10 min at 95°C, the mixture was amplified in cycles of 30 s at 95°C, 30 s at 59°C, and 1 min at 72°C. Size and specificity of the products were verified by agarose gel electrophoresis. Product abundance relative to controls was calculated assuming linearity to log (initial copies). Primers are given in Table 1.

**Table 1.** Mouse primers

ALP (alkaline phosphatase)—Product 131 bp
F: 5′- ATCGGAACAACCTGACTGACCCTT-3′ R: 5′- ACCCTCATGATGTCCGTGGTCAAT-3′
ANK (ANKH, progressive ankylosis)—Product 132 bp
F: 5′- ATGGTGAAATTCCCGGCGCTCAC-3′ R: 5′- CATCCTCCTTGACTGCAGCGATG-3′
BGLAP (osteocalcin)—Product 118 bp
F: 5′- ACCATCTTTCTGCTCACTCTGCTG-3′ R: 5′- TATTGCCCTCCTGCTTGGACATGA-3′
Col1a1 (collagen type 1)—Product 159 bp
F: 5′- TTCTCCTGGCAAAGACGGACTCAA-3′ R: 5′- AGGAAGCTGAAGTCATAACCGCCA-3′
CLCN3 (H^+^/Cl^−^ exchanger)—Product 112 bp
F: 5′- CCAAGACCCCGCTTCAATAA-3′ R: 5′- CGAGTCCCGCAGATTAAAGA-3′
ENPP1 (ecto-nucleotide pyrophosphatase/phosphodiesterase 1)—Product 207 bp
F: 5′- CGCCACCGAGACTAAA-3′ R: 5′- CGGACGCTATGATTCCT-3′
ENPP2 (ecto-nucleotide pyrophosphatase/phosphodiesterase 2)—Product 71 bp
F: 5′- TGGCTTACGTGACATTGAGG-3′ R: 5′- AGTGGGTAGGGACAGGAATAG-3′
NPT2 (type 2 sodium-phosphate cotransporter)—Product 127 bp
F: 5′- GGCTCCAACATTGGCACTAC-3′ R: 5′- ACAGTAGGATGCCCGAGATG-3′
Smad1 (suppressor of mothers against decapentaplegic 1)—Product 136 bp
F: 5′- CTCATGTCATTTATTGCCGTGTG-3′ R: 5′- CGCTTATAGTGGTAGGGGTTGA-3′
Smad2 (suppressor of mothers against decapentaplegic 2)—Product 173 bp
F: 5′- ATGTCGTCCATCTTGCCATTC-3′ R: 5′- AACCGTCCTGTTTTCTTTAGCTT-3′
β-catenin [CTNNB1, catenin (cadherin associated protein), beta 1]—Product 146 bp
F: 5′- GTTCGCCTTCATTATGGACTGCC-3′ R: 5′- ATAGCACCCTGTTCCCGCAAAG-3′
GAPDH (glyceraldehyde-3-phosphate dehydrogenase)—Product 184 bp
F: 5′- GTTGTCTCCTGCGACTTCA-3′ R: 5′-GGTGGTCCAGGGTTTCTTA-3′

### Assays of Alkaline Phosphatase Activity and von Kossa Mineral Staining

Alkaline phosphatase activity was determined using 7-bromo-3-hydroxy-2-naphthoic-O-aniside (naphthol AS-BI phosphate) substrate, reacted with fast blue to precipitate blue insoluble product, at pH 9.5 (leukocytes alkaline phosphatase kit, Sigma). Matrix mineralization was evaluated using silver nitrate (von Kossa) staining (9). Digital images were acquired by scanning plates at 600 dpi (dots per inch) using a Umax Power Look III Color Scanner Flatbed Scanner. Cultures were imaged to show nodular bone formation.

### Western Blots

Cells were lyzed on ice in RIPA extraction buffer (Thermo Fisher) with proteinase and phosphatase inhibitors. Lysates were sonicated and cleared by centrifugation. Then 4X Laemmli buffer with 2-mercaptoethanol was mixed with 25 µg of protein. Samples were heated for 5 min at 70°C, briefly centrifuged, and loaded onto 4%–12% Bis-Tris protein precast gels (Thermo Fisher). Gels were run in 3-(*N*-morpholino) propane sulfonic acid buffer; proteins were transferred to polyvinylidene difluoride membranes (Bio-Rad, Hercules, CA) by semidry transfer (Bio-Rad). The membranes were rinsed in tris-buffered saline with Tween-20 buffer and blocked for 2 h in 5% blocking reagent (Bio-Rad). After blocking, the primary antibody (diluted in blocking solution) was added to the membranes and incubated overnight at 4°C. ERK1/2 and Phospho-Erk1/2 monoclonal rabbit Ab (1:200, Cell Signaling), ALP polyclonal goat Ab (at 1 µg/mL, R&D Systems), or β-actin monoclonal mouse Ab (1:10,000, Sigma) were used. Unbound antibody was removed by washing. Labeled proteins were detected with peroxidase-linked secondary antibody (1:10,000, Jackson ImmunoResearch) for 1 h at room temperature. Blots were stripped and reprobed. Bound antibodies were visualized by enhanced chemiluminescence (SuperSignal West Femto Maximum, Thermo Fisher) and detected using a gel doc system (ChemiDoc, Thermo Fisher). Protein intensity was quantified using ImageJ software (NIH; public domain).

### Micro-computed Tomography to Verify Bone Loss in 18- to 19-Mo-Old Animals

Micro-computed tomography (microCT) used lumbar vertebra L4 and L5. Bone samples were fixed overnight in 3.7% formalin, switched to 70% ethanol, and kept at −20°C until used. MicroCT analysis used a 1172 SkyScan (Bruker, Allentown, MA) at 6 µm resolution with a 0.5-mm aluminum filter. Images of L4 and L5 were reconstructed separately and analyzed using the NRecon, CT analyzer, and CTvox software; cross-sectional images used Data Viewer (Bruker).

### Statistics

Statistical significance of data was evaluated with the Student *t* test (*P* < 0.05). Data are reported as means ± standard deviation.

## RESULTS

### Major Bone Matrix and Mineral Transport mRNAs Decrease in Marrow Cells of Old Mice In Vivo

**Figure 1.**
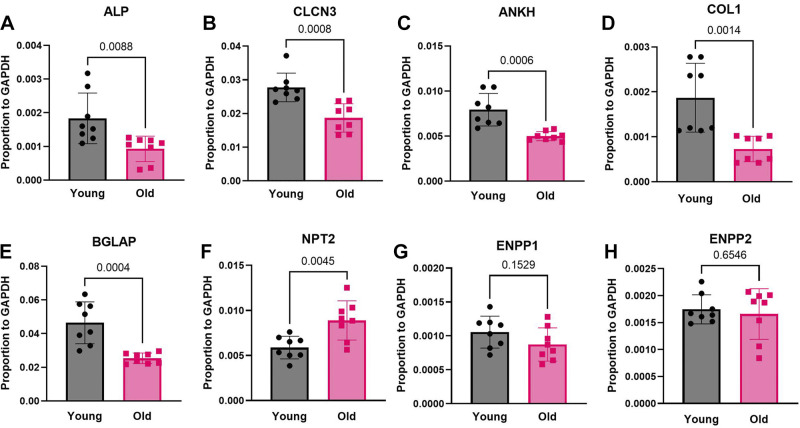
Real-time-PCR analysis of mRNA expression in bone marrow cells from trabecular bone surfaces and bone marrow of 3- or 4-mo-old and 18 -or 19-mo-old mice. Young indicates 3or 4 mo and old indicates 18 or 19 mo mice. *n* = 8, 4 males and 4 females. The statistical significance of data was evaluated with the Student *t* test. *A*: alkaline phosphatase. *B*: ClC3 chloride/hydrogen exchanger. *C*: ANKH pyrophosphate-associated membrane protein. *D*: type I collagen. *E*: bone Gla (osteocalcin) mRNA. *F*: neutral phosphate transporter-2. *G*: ectonucleotide pyrophosphatase/phosphodiesterase 1. *H*: ectonucleotide pyrophosphatase/phosphodiesterase 2.

Initial quantitative assays used PCR of cells flushed from marrow of young and old mice, directly used for reverse transcription and real-time PCR (methods). The data, shown in Fig. 1, were normalized to GAPDH to assure that changes in mRNA were controlled overall. Specifically, mRNAs for the alkaline phosphatase decreased ∼30%, *P* = 0.009; for the ClC3 chloride/hydrogen exchanger decreased ∼30%, *P* = 0.0008; and for the ANKH pyrophosphate-associated membrane protein decreased ∼25%, *P* = 0.0006. Furthermore, mRNAs for type I collagen decreased ∼50%, *P* = 0.0014 and for the bone Gla protein decreased ∼40%, *P* = 0.0004. The mRNAs for three transport proteins were not decreased with age, neutral phosphate transporter-2, which actually increased ∼30%, *P* = 0.004 and for the two ectonucleotide pyrophosphatase/phosphodiesterases 1 and 2, not significant (Fig. 1). Note that the cells used contain osteoblasts, but also other cells, and the clearest results, including alkaline phosphatase and type I collagen, are highly expressed in osteoblasts.

#### Limitations of these data.

**Figure 2.**
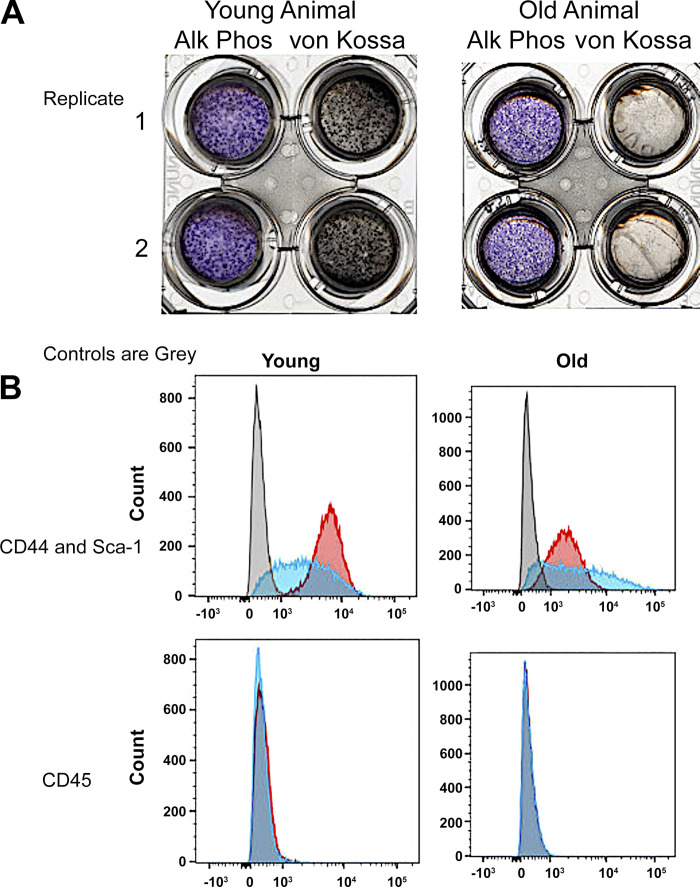
Characterization of stromal stem cells and osteoblasts. *A*: examples of alkaline phosphatase and von Kossa stains for 2 replicates out of 4 young (2 males and 2 females) and 4 old (2 males and 2 females) mice. Each well is a whole well from a 6-well plate with the inner part of the image the perforated polyethylene terephthalate membrane containing the complete ∼1.6-cm wide cell culture. The images shown are from 3-wk differentiated cultures, where transepithelial resistance is ∼1,400 Ω-cm (9). *B*: flow cytometry showing that preparations essentially uniformly expressed CD44 and Sca1, but not CD45 (negative control). Gray bars are isotype controls. Example are shown; there was no meaningful differences in flow analysis of any SSC culture out of 4 young (2 males and 2 females) and 4 old (2 males and 2 females) mice. SSC, stromal stem cell.

All of these proteins are widely distributed except, perhaps, the pyrophosphate enzymes. Since specific mRNA expression varied, it seemed appropriate to test expression in homogenous osteoblast cultures. Some proteins including ClC3 and the neutral phosphate transporter 2 are expressed in many cells and there is no assurance that the mRNA reflects osteoblast expression. These data, although interesting, were seen as preliminary and were supplanted by more specific studies using osteoblasts made from stromal stem cells on perforated polyethylene terephthalate membranes (9) (Fig. 2).

### Differentiation of Osteoblasts from Stromal Stem Cells

**Figure 3.**
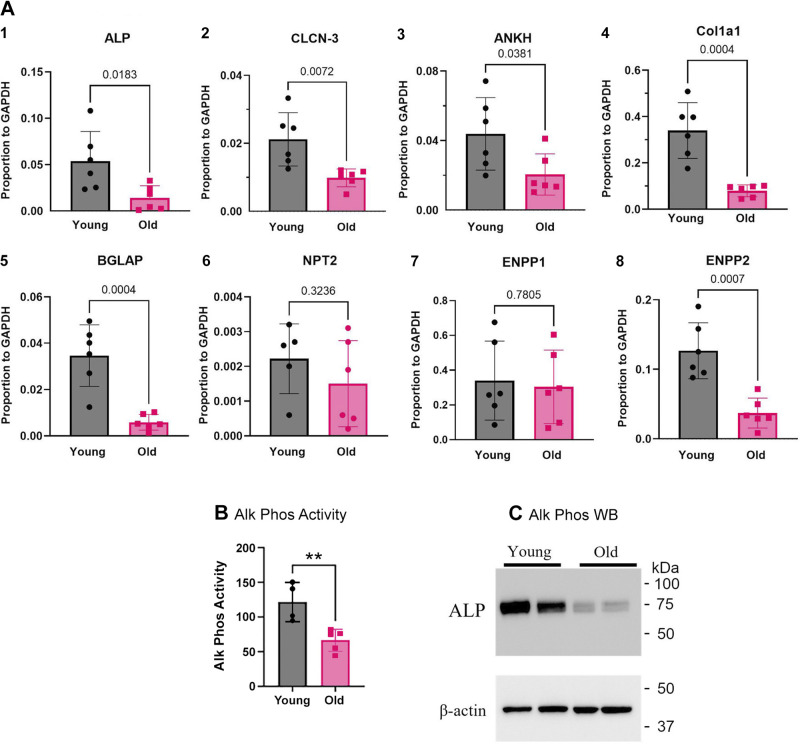
Analysis of transport proteins in cells differentiated on PET membranes, including activity and Western blot for alkaline phosphatase. *A*: real-time PCR analysis of mRNA expression in osteoblasts from 6 young (3 males and 3 females) and 6 old (3 males and 3 females) mice differentiated for 2 wk on PET membranes. The statistical significance of data was evaluated with the Student *t* test. Data are similar to Fig. 1, with less variability in most cases. In one case (ENPP2), a difference is resolved, not seen in bone marrow cells; see text. *1*) Alkaline phosphatase; *2*) ClC3 chloride/hydrogen exchanger; *3*) ANKH pyrophosphate-associated membrane protein mRNA; *4*) type I collagen; *5*) bone Gla (osteocalcin) mRNA; *6*) neutral phosphate transporter-2; *7*) ectonucleotide pyrophosphatase/phosphodiesterase 1; 8. Ectonucleotide pyrophosphatase/phosphodiesterase 2. *B*: alkaline phosphatase activity. See Fig. 2*A* for images. *P* < 0.01, *n* = 4. *C*: Western blots for alkaline phosphatase in young and old cultures. ALP, alkaline phosphatase; ENPP, ecto-nucleotide pyrophosphatase/phosphodiesterase; PET, polyethylene terephthalate.

Characterization of osteoblasts differentiated from stromal cells is illustrated in Fig. 2. Examples of alkaline phosphatase and von Kossa stains for two replicates of individual preparations from young and old mice at 3 wk of differentiation are shown in Fig. 2*A*; this was done for all preparations. For comparison of alkaline phosphatase activity between preparations and Western blots, see Fig. 3.

Occasional cultures failed for various reasons and were discarded, these included lack of electrical resistance across cell layers and lack of alkaline phosphatase labeling. Figure 2*B* shows flow analysis of preparations for stem cells, CD44 and Sca1, and a control antigen, CD45. The SSCs were produced with special media for stem cell isolation (methods). Preparations are essentially uniformly positive for CD44 and Sca1, and negative for CD45. In Fig. 2*B*, the gray bars are negative controls. For additional characterization of the osteoblast cultures, including electron microscopy, the electrical resistance of epithelial-like osteoblast layers, and pH gradients across epithelial-like osteoblast layers see Ref. 9.

### Bone Matrix Proteins and Mineral Transporter mRNA Expression in Osteoblasts Differentiated on PET Membranes

To isolate effects from marrow cells that include macrophages and osteoclasts, we repeated this study in osteoblasts differentiated on PET membranes in vitro. Bone matrix proteins and mineral transporters mRNA expression measured by real-time PCR (Fig. 3) decreased with age in essentially the same way as effects in vivo in mixed cell preparations (Fig. 1), but in most cases with larger average changes, and in some cases, significant differences were seen that were not detected in marrow preparations. Data were normalized to GAPDH as in marrow preparations. In detail, mRNAs for the alkaline phosphatase decreased in old mice ∼80%, *P* = 0.02, for the ClC3 chloride/hydrogen exchanger decreased ∼50%, *P* = 0.007, the ANKH pyrophosphate-associated membrane protein decreased ∼50%, *P* = 0.04. Furthermore, mRNAs for type I collagen and bone Gla protien were decreased ∼75%, both with *P* = 0.004. The mRNA for ectonucleotide pyrophosphatase/phosphodiesterase 2 was reduced in osteoblasts from older animals by ∼75%, *P* = 0.0007. On the other hand, mRNAs for two transport proteins were not significantly changed with age, neutral phosphate transporter-2 and ectonucleotide pyrophosphatase/phosphodiesterase 1.

### Trabecular Bone and Its Morphometric Parameters Decline with Age Consistent with Changes in Mineral Transport Proteins

**Figure 4.**
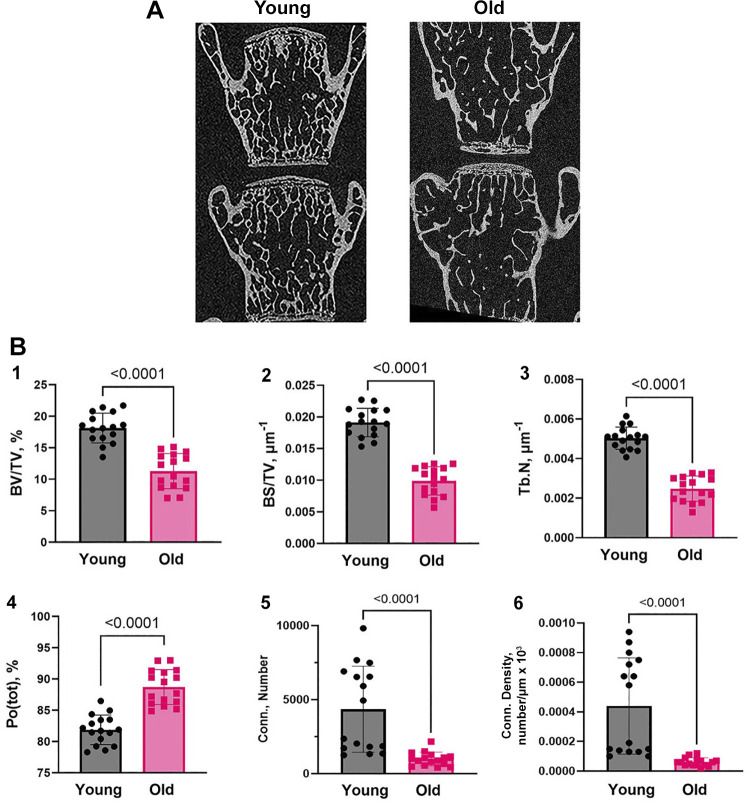
MicroCT analysis of lumbar vertebra (L4 and L5) from 4 young and 4 old mice. *A*: microCT images of lumbar vertebra L4 and L5 from young (3 or 4 mo) and old (18 or 19 mo) mice. *B*: *1*) percent bone volume, *2*) bone surface density, and *3*) trabecular number are significantly decreased; *4*) total porosity, and is significantly increased in old mice. Two measures of connectivity *5*) connectivity and *6*) connectivity density are significantly decreased in old mice. In each case, *P* < 0.0001, *n* = 16, vertebra L4 and L5 were analyzed in 4 young (2 males + 2 females) and 4 old (2 males + 2 females) mice. The statistical significance of data was evaluated with the Student *t* test.

MicroCT images of lumbar vertebra L4 and L5 from young and old mice showed that trabecular bone of the old mice is osteopenic relative to the young mice (Fig. 4*A*). This is in keeping with other studies, but necessary to document that the populations used have significant age-dependent bone loss. Trabecular bone morphometry parameters (Fig. 4*B*), including percent bone volume, bone surface density, and trabecular number, were reduced in old mice by about 40%–50% and *P* < 0.001. As expected with lower density, porosity increased with age approximately doubling, and two measures of connectivity, connectivity and connectivity density, decreased about 75% in old mice. These also had *P* < 0.001 between groups. In all cases, *n* = 8. Since our focus is solely on bone formation, we did not study bone resorption or osteoclast surface.

### Osteoblast Differentiation Is Downregulated in Old Mice through the TGFβ and WNT/β-Catenin Pathways

**Figure 5.**
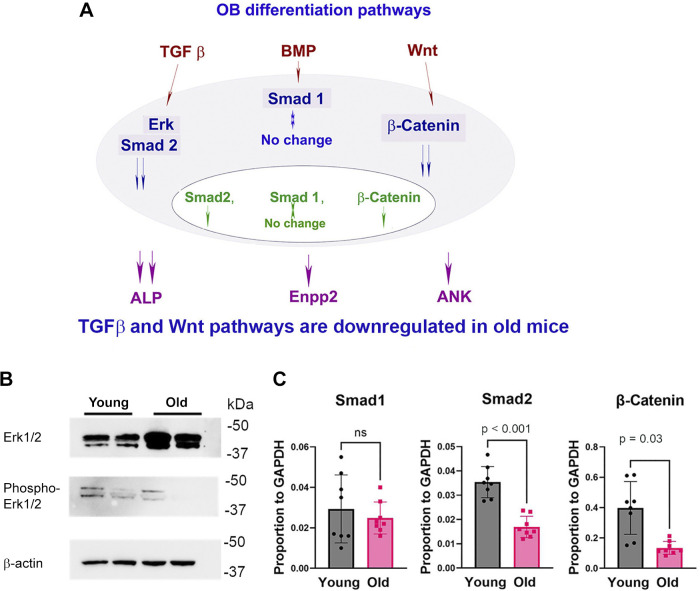
Age-dependent downregulation of OB differentiation is associated with the TGFβ and WNT/β-catenin pathways. *A*: OB differentiation pathway map. OBs were differentiated from SSC on PET membrane for 2 wk. *B*: age-dependent decrease in Erk1/2 phosphorylation. With *n* = 2, difference of phosphoErk/Erk was ∼80%, *P* = 0.02. *C*: Smad1, Smad2, and β-catenin RNA expression. Changes in Smad1 were not significant; Smad2 and β-catenin decreased 50% and 75% and were significant at *P* < 0.001 and *P* = 0.03, respectively; *n* = 8 (4 males and 4 females). The statistical significance of data was evaluated with the Student *t* test. OB, osteoblast; PET, polyethylene terephthalate; SSC, stromal stem cell.

Pathway analysis of key mechanisms in differentiation was investigated in osteoblasts differentiated on PET membrane for 2 wk. This is particularly interesting in light of findings to this point, suggesting that differences in osteoblast differentiation with age are durable (see discussion). A differentiation pathway map of common mechanisms is given in Fig. 5*A*. Age-dependent decrease in Erk1/2 phosphorylation is shown in Fig. 5*B*. Erk1/2 phosphorylation is related to the transforming growth factor-β (TGFβ) pathway. With *n* = 2, difference of phosphoErk/Erk was ∼80%, *P* = 0.02. One of two assays with similar findings is shown. The mRNA for suppressor of mothers against decapentaplegic 1 (Smad1), related to the bone morphogenetic protein (BMP) pathway, was not changed. In contrast, mRNA for Smad2 and β-catenin decreased in aged mice with *P* < 0.001 and *P* = 0.03, respectively (Fig. 5*C*). Decreased Smad2 mRNA is consistent with TGFβ pathway regulation. Decreased β-catenin is related to the Wingless-related integration site (WNT) signaling pathway of osteoblast differentiation. In addition, downregulated ALP activity and mRNA in osteoblasts of old mice is an important association of osteoblast differentiation with BMP, this is shown in Figs. 2 and 3.

## DISCUSSION

This work, for the first time, determined proteins for bone mineral transport and matrix synthesis that are regulated in osteoblasts as a function of age, when bone synthesis decreases. We initially used real-time PCR to evaluate mineral transport and matrix protein transcription in cells from bone marrow of young (3 or 4 mo) and old (18 or 19 mo) mice (Fig. 1). Although the cells are not pure, the mRNAs are largely specific osteoblast products, and this showed interesting differences. Specifically, old animals made less of the proteins, implicated in bone mineral production (5–7). Individual proteins are discussed in more detail, and in defined cell preparations in *Proteins That Varied With Age*, below. This was accomplished using osteoblasts differentiated from stromal stem cells (Fig. 2). These cells, also called skeletal stem cells, and, formerly, mesenchymal stem cells are produced from bone marrow cells using highly selective media.

Differentiation of SSC to produce osteoblasts in vitro was characterized by alkaline phosphatase and mineral assays (Fig. 2*A*) on perforated polyethylene terephthalate membranes (9). The nature of the SSC was verified by flow analysis for stem cell and control markers (Fig. 2*B*).

When osteoblasts from the cultures on PET membranes were assayed for the bone mineral transport and matrix proteins, very clear results were obtained for the target enzymes (Fig. 3). This is most interesting from the point of view that changes with age in stem cells were durable, or, in other words, these stem cells are memory cells, with those differences in old animals maintained during in vitro differentiation (Fig. 2) and obviously correlating with direct real-time PCR from cells flushed out of the marrow (Fig. 1). In addition, most, but not all, of the enzymes involved in mineral production were reduced in older animals. Specifically, mRNAs for alkaline phosphatase, the ClC3 chloride/hydrogen exchanger, the ANKH pyrophosphate-associated membrane protein, implicated in bone mineral production (5–7), as well as the ectonucleotide pyrophosphatase/phosphodiesterase 2 (ENPP2) and structural proteins type 1 collagen and bone gla protein were reduced.

### Proteins That Varied with Age

A survey of the literature shows how the proteins regulated function in bone formation, but provide limited insights into why these proteins are coordinated with age. In brief, the ankylosis protein (ANKH or ANK) may transport PPi (pyrophosphate), ATP, or citrate, with variation in findings between several studies (19–21). ANKH mRNA in osteoblasts grown on membranes was reduced with age in SSC. For ClC3, the reduction in mRNA expression in old mice is keeping with our earlier work, showing a requirement of ClC3 and ClC5 for mineralization in vitro (5). This is controversial in that ClC3 or ClC5 is usually seen as endosomal, or intracellular, enzymes involved in vesicle transport; this is not the place to discuss the much higher expression of these proteins in osteoblasts. It is worth noting, however, that other work indicates roles for ClC3, ClC4, and ClC5 in osteoblast mineral production (22). Collagen 1 is reported to be downregulated with age in cortical bone cells by RNA-seq (23). Alkaline phosphatase may be, at least in part, an exception to regulation of age dependence, in that ablation of ALP in the bone was shown to induce premature aging, and might be a candidate for overall regulation of stemness (24). This deserves additional analysis. The role of ENPP2 in mineralization remains to be evaluated. In contrast to ENPP1, ENPP2 expression was suppressed in osteoblasts from old animals (Fig. 3). ENPP2 was not previously identified in studies of mineral transport and bone formation (4–7).

ENPP2 expression has been reported in muscle, osteo/chondrogenic, and tooth development (25–26). Expression of ENPP2 was previously shown to be downregulated in osteoblasts differentiated in acidic conditions and to be related to Wnt/β-catenin signaling (25, 27), but the role of ENPP2 in bone formation was not described. Furthermore, direct analysis on the role for ENPP2 in osteoblast differentiation is planned for further work.

Proteins that did not vary with age included the neutral phosphate transporter-2, which imports phosphate into osteoblasts (17), and the ectonucleotide pyrophosphatase/phosphodiesterase 1 (ENPP1). Neutral phosphate transport is an overall regulator of Pi (phosphate) homeostasis required for skeletal development (28), but it has no clear role in reduced bone formation with aging.

Unsurprisingly, the *Npt2* knockout mouse has poorly developed metaphyseal trabecular bone and retarded secondary ossification at 21 days, which is reversed and overcompensated at ∼3 mo (28). Another important basolateral (marrow side) transport protein, the sodium-hydrogen exchanger NHE1 (4), was unchanged with age and was not further studied. Ectonucleotide pyrophosphatase/phosphodiesterases are interesting but will need further study. In brief, ENPP1 has been implicated in osteoblast differentiation (29) by a mechanism independent of catalysis (30). Our data neither confirm nor oppose this. ENPP1 is a transmembrane ectoenzyme known to affect bone formation in vitro in MC3T3E1(C4) osteoblast-like cells (29), and, importantly, in other contexts than bone differentiation, it is involved in regulating calcification of other tissues including arterial walls (31).

Furthermore, regarding the memory of age effects on osteoblasts differentiated from stromal stem cells, differences with age of animals from which SSCs were derived clearly extended to regulatory genes for osteoblast differentiation in the TGFβ and WNT/β-catenin pathways (Fig. 5). Pathway analysis showing that age-dependent downregulation of osteoblast differentiation involves Smad2-dependent TGFβ and Wnt/β-catenin signaling pathways is not surprising based on multiple studies of these pathways in bone (32–33), but indicates that changes in bone formation with age are not independent of bone gene expression pathways otherwise defined. Decreased ALP expression in osteoblasts from old mice might be associated with downregulation of the BMP, TGFβ, and Wnt/β-catenin signaling pathways (34–35). Osteopenia in the old mice (36) was validated as an important control for the work (Fig. 4).

### Perspectives and Significance

**Figure 6.**
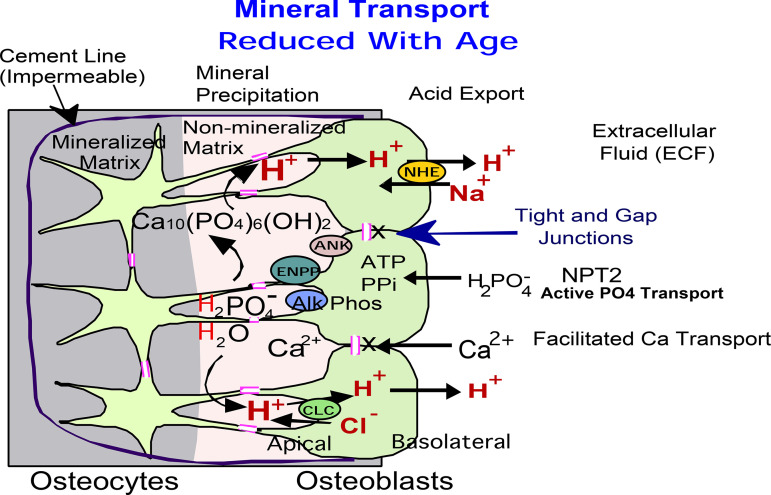
Hypothetical model of bone formation pathway in osteoblasts. This model is for bone formation only and does not include bone degradation, not studied. Our results indicate that age-dependent decline in trabecular bone correlates with downregulation of expression of major bone matrix proteins, collagen 1 and osteocalcin, together with PPi-related alkaline phosphatase (ALP), ecto-nucleotide pyrophosphatase/phosphodiesterase (ENPP2), ankylosis (ANK) proteins, and Cl^−^/H^+^ antiporter ClC3. These proteins are located on the apical (bone forming) side of the epithelial-like layer of osteoblasts connected by tight and gap junctions and are responsible for the mineral and ion transport between cytoplasm and extracellular matrix. This is hypothetical in the case of ClC3, better known as an endosomal protein. Basolateral proteins, on the extracellular fluid side of the osteoblast epithelium-like layer (NPT2 and NHE1), appear not to be significantly regulated with age.

Mineral-producing proteins are diagrammed in a hypothetical model (Fig. 6), those that change with age are apical (bone interface) and those that do not change with age are, particularly NPT2, on the opposite, basolateral surface. Our results indicate that age-dependent decline in trabecular bone structure correlates with durable (also called memory) changes in expression of major bone-producing proteins, demonstrated using osteoblasts differentiated from stromal stem cells from young and old C57Bl6 mice. Overall, our work confirms the relation of mineral transport with reduction of bone mass with aging, and points to changes with aging in the potential of the stem cells that produce bone, or memory. This is a subject of interest with several related publications (37), but as yet, a clear mechanism responsible for memory is not known.

## DATA AVAILABILITY

Data will be made available upon reasonable request.

## GRANTS

The National Institutes of Health, National Institute on Aging provided old mice. Funded in part by BX002490-06A1 from the Department of Veteran’s Affairs to H.C.B., and by R01 AR076146-01 from the National Institutes of Health to H.C.B.

## DISCLOSURES

No conflicts of interest, financial or otherwise, are declared by the authors.

## AUTHOR CONTRIBUTIONS

Q.C.L., S.L., J.L., P.H.S., and H.C.B. conceived and designed research; I.L.T., Q.C.L., K.M.O., S.L., and H.C.B. performed experiments; I.L.T., Q.C.L., and H.C.B. analyzed data; I.L.T., Q.C.L., S.L., J.L., P.H.S., and H.C.B. interpreted results of experiments; I.L.T., Q.C.L., K.M.O., and H.C.B. prepared figures; I.L.T. and H.C.B. drafted manuscript; P.H.S. and H.C.B. edited and revised manuscript; I.L.T., K.M.O., S.L., J.L., P.H.S., and H.C.B. approved final version of manuscript.
